# Rectal endoscopy findings following stereotactic body radiation therapy for clinically localized prostate cancer

**DOI:** 10.1186/1748-717X-8-197

**Published:** 2013-08-09

**Authors:** Sumit Sood, Andrew W Ju, Honkung Wang, Siyuan Lei, Sunghae Uhm, Guowei Zhang, Simeng Suy, John Carroll, John Lynch, Anatoly Dritschilo, Sean P Collins

**Affiliations:** 1 Department of Radiation Medicine, Georgetown University Hospital, LL Bles Building, 3800 Reservoir Rd. NW, Washington, DC 20007, USA; 2 Department of Biostatistics and Bioinformatics, Georgetown University, Washington, DC, USA; 3 Department of Gastroenterology, Georgetown University Hospital, Washington, DC, USA; 4 Department of Urology, Georgetown University Hospital, Washington, DC, USA

**Keywords:** Stereotactic body radiotherapy, Prostate cancer, SBRT, CyberKnife, Hypofractionation, Endoscopy, Rectal toxicity, Quality of life

## Abstract

**Background:**

Treating prostate cancer with SBRT could potentially minimize radiation proctitis by reducing high-dose rectal irradiation. In addition, it offers the potential radiobiologic benefits of hypofractionation. This study reports the endoscopic changes and the associated clinical rectal toxicity in these patients.

**Methods:**

We reviewed the records of patients treated from 2008–2011 for localized prostate cancer who had rectal endoscopy following SBRT. SBRT was delivered either as primary treatment in 5 fractions of 7–7.25 Gy, or as an initial boost in 3 fractions of 6.5 Gy followed by conventionally fractionated radiotherapy to 45–50.4 Gy. Endoscopic changes were graded using the Vienna Rectoscopy Score (VRS). Rectal toxicity was graded via CTCAEv.4. Rectal quality of life (QOL) was assessed via the bowel domain of the EPIC-26 questionnaire.

**Results:**

Fifty-one patients with a median 23 months follow-up were analyzed. Thirty-five patients completed SBRT monotherapy and 16 patients received SBRT as a boost to conventionally fractionated IMRT. The median interval from SBRT to rectal endoscopy was 13 months. Endoscopy revealed VRS Grade 1–2 telangiectasias for 10 patients and VRS Grade 1–2 mucosal edema for 12 patients. No rectal ulcerations, strictures or necrosis were observed. Grade 1–2 late rectal bleeding occurred in 10 patients. There were no CTCAEv.4 Grade ≥3 toxicities. Mean EPIC bowel scores decreased from a baseline value of 96.9 to 82.3 at 1-month, but subsequently increased to 91.0 at 24 months.

**Conclusions:**

In this cohort that is skewed towards patients with rectal complaints, the rate and severity of endoscopic changes following SBRT is low. Rectal toxicity and QOL were comparable to patients treated with other radiation modalities. Prospective trials examining the endoscopic outcomes following SBRT for prostate cancer are needed for confirmation of the findings of this study.

**Trial registration:**

The Georgetown Institutional Review Board has approved this retrospective study (IRB 2009–510).

## Background

Radiation therapy is a well-established treatment modality for clinically localized prostate cancer. Late radiation proctitis occurs at a frequency of 5–20% when radiotherapy is delivered with conventional radiation therapy for localized prostate cancer [1]. Patients with radiation-induced proctopathy describe symptoms of rectal pain, bowel frequency/urgency and rectal bleeding. These symptoms occur months to years after treatment (average 8–12 months), with the large majority of patients reporting symptoms within two years following pelvic radiation therapy [2,3]. Patient characteristics such as a history of hemorrhoids, inflammatory bowel disease [4] or anticoagulation therapy [5] may increase an individual patient’s risk for clinically significant proctopathy. Endoscopic findings in patients with clinical proctopathy include telangiectasia, congested mucosa, and ulcers. Rectal bleeding from neovascular telangiectasias is observed in 20-88% of patients receiving conventionally fractionated radiation therapy [6]. These rectal complications are the principle dose-limiting toxicities of radiotherapy. Several trials have demonstrated an improved biochemical failure-free survival with dose-escalation, but the increased rates of rectal toxicities are a potential barrier to the use of escalated doses [7-9].

The risk of proctitis and rectal bleeding appeared to be dependent upon both the total radiation dose and the volume of the rectum in the high dose area [10]. Treatment-related factors such as prostate motion and radiation schedules can contribute substantially to the severity of rectal toxicities. The prostate gland has been shown to move both interfractionally and intrafractionally during the delivery of external-beam radiotherapy [11]. As a result, a 0.5-1.5 cm margin is usually added to the clinical target volume (CTV) in generating the planning treatment volume (PTV) to account for this motion with conventional radiotherapy or intensity-modulated radiotherapy (IMRT). The need for such margins limits the ability to escalate dose to the prostate and spare normal tissues.

The optimal radiation schedule for the curative treatment of prostate cancer remains unknown. Recent data suggest that large radiation fraction sizes are radiobiologically favorable over lower fraction sizes in prostate cancer [12-14]. The α/β for prostate cancer may be as low as 1.5 Gy, [14] as opposed to values of 6–8 Gy reported for other adenocarcinomas [12]. If the α/β for prostate adenocarcinoma is less than the value of 3 Gy that is generally accepted for late rectal complications, the linear-quadratic model predicts a greater therapeutic gain for hypofractionated radiotherapy over conventionally fractionated treatment regimens. High dose-rate (HDR) brachytherapy using 6–9.5 Gy per fraction has been shown to be safe and effective in the treatment of localized prostate cancer [15-17]. The use of large fraction sizes in SBRT offers the potential radiobiologic benefits of hypofractionation with the minimal invasiveness of an external-beam treatment modality.

Stereotactic body radiation therapy (SBRT) offers to minimize radiation-associated rectal toxicity by reducing the volume of rectum receiving high radiation doses. The CyberKnife robotic radiosurgical system uses image guidance to track implanted fiducials to account for intrafraction prostatic motion [18]. This decreases the uncertainty of the location of the prostate and allows treatment to be delivered with a smaller CTV to PTV expansion, which reduces the doses delivered to the rectum. Early results from our center [19] and others [20-22] suggest a similar efficacy as alternative radiation modalities with low rates of late Grade ≥2 rectal toxicity (< 10%). The goal of this study is to report the endoscopic findings following SBRT for clinically localized prostate cancer and correlate these outcomes with clinical rectal toxicity and quality of life.

## Methods

Patients treated at our institution with SBRT for clinically localized prostate cancer who underwent at least one post-treatment rectal endoscopy were identified and included in this retrospective review. Clinical stage was defined according to the 6^th^ edition of the American Joint Committee on Cancer criteria. Risk groups were defined using the National Comprehensive Cancer Network (NCCN) criteria. Institutional review board approval was obtained for this review.

**Figure 1:**
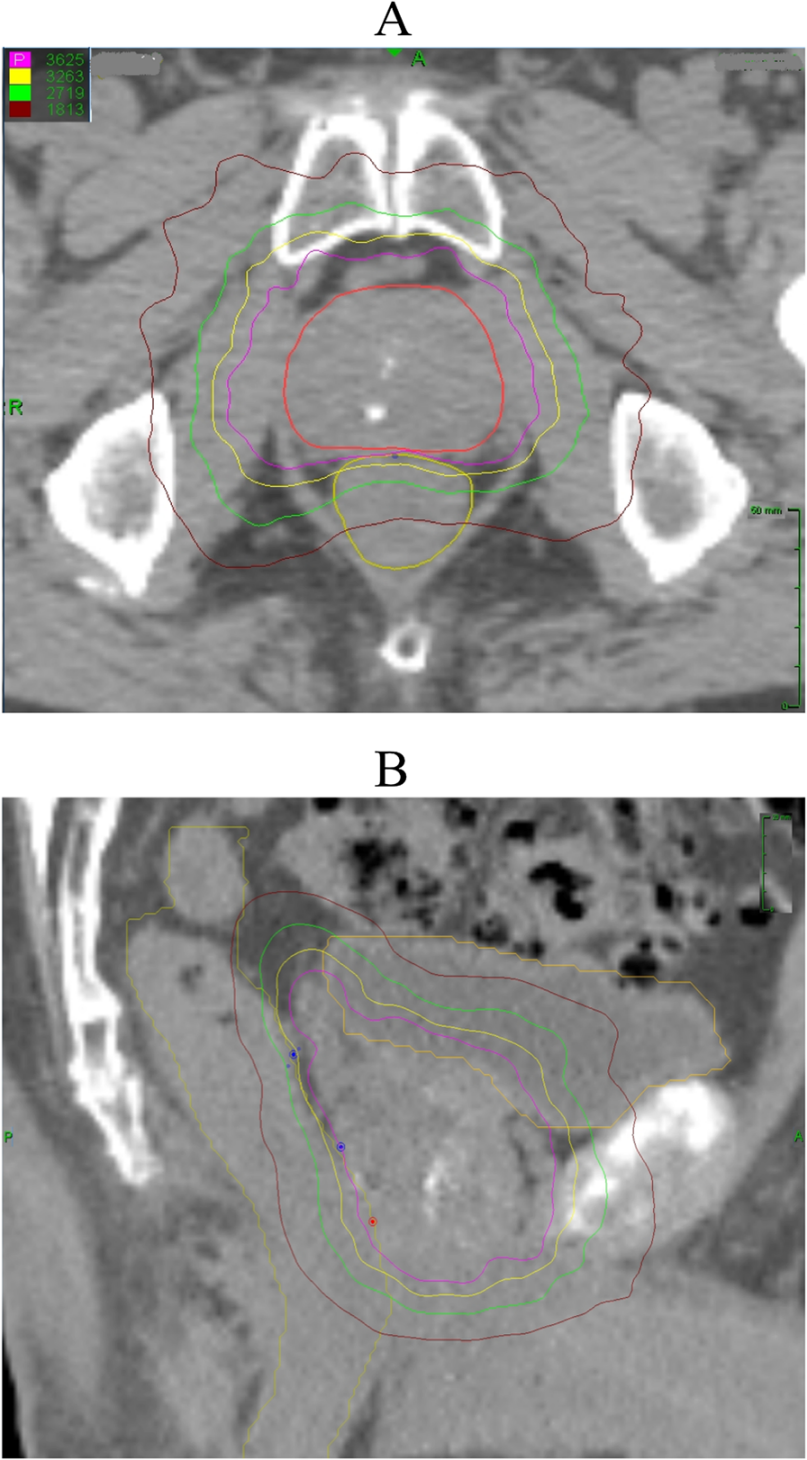
**Treatment planning scan.** Axial **(A)** and sagittal **(B)** computed tomography images the prostate GTV (red line), bladder (orange line) and rectum (yellow demonstrating line). Isodose lines shown as follows: 100% of the prescription dose (lavender line); 90% of the prescription dose (yellow line); 75% of the prescription dose (green line); 50% of the prescription dose (brown line).

SBRT was delivered using the CyberKnife robotic radiosurgical system. The fiducial placement and CT/MRI simulation procedures have been previously described in Lei *et al*. [23]. The clinical target volume (CTV) was defined as the prostatic capsule and proximal seminal vesicles (SV) up to the point that the SVs split, and included gross extracapsular extension or SV involvement seen on MRI. The expansion from the CTV to the planning target volume (PTV) was 5 mm in all directions except 3 mm posteriorly into the rectum. Fiducial-based tracking was used to account for intrafraction and interfraction prostate motion. Treatment planning was performed using Multiplan (Accuray Inc., Sunnyvale, CA). Patients with low-risk prostate cancer and select patients with intermediate-risk cancer were treated with 35 or 36.25 Gy of radiotherapy delivered in 5 fractions of 7–7.25 Gy each to the PTV [21,24]. Patients with high-risk prostate cancer and most patients with intermediate-risk cancer were treated with 19.5 Gy of radiotherapy delivered in 3 fractions of 6.5 Gy each as a boost, followed by 45–50.4 Gy of intensity-modulated radiotherapy (IMRT) delivered in 1.8 Gy daily fractions [25]. The dose constraints to the rectum for SBRT have been previously described [24,25]. An example of the dose distributions from an SBRT monotherapy plan is shown in Figure 1. Patients were placed on a low-residual diet and given enemas prior to simulation and treatment delivery to maximize the potential distance between the prostate and the rectal wall and minimize intrafraction prostate motion.

Endoscopic proctopathy was graded and scored using the Vienna Rectoscopy Score (VRS) [26]. For VRS telangiectasia grading, a single telangiectasia is Grade 1, multiple non-confluent telangiectasia Grade 2, and multiple confluent telangiectasia Grade 3. For VRS congested mucosa grading, focal reddening of the mucosa combined with edematous mucosa is Grade 1, diffuse non-confluent reddening of the mucosa combined with edematous mucosa Grade 2, and diffuse confluent reddening of the mucosa combined with edematous mucosa Grade 3. If a patient had more than 1 endoscopy in the follow-up period, the worst endoscopic findings seen between all studies were recorded.

Clinical rectal toxicities were prospectively graded at each visit via CTCAE v.4. Clinical acute toxicities were defined as an increase of symptoms over the patient’s pre-treatment baseline at any follow up visit prior to or at 6 months and late toxicities were defined as those that occur after 6 months. The toxicities analyzed were bowel frequency/urgency, rectal pain and rectal bleeding. At each follow-up visit, toxicity events were scored independently for each of the different toxicity types and the highest GI toxicity was determined for each patient. Quality of life (QOL) was assessed via the bowel domain of the Expanded Prostate Cancer Index (EPIC)-26 questionnaire prior to treatment and at scheduled follow-up visits after completion of radiotherapy [27]. The symptoms assessed included bowel urgency, bowel frequency, rectal incontinence, rectal bleeding, and rectal pain. As part of our institutional practice, if patients were unable to return to our institution for follow-up, QOL questionnaires were mailed out at the time points where the patient would normally have been seen, and the clinic notes from their follow-up visits with local physicians were obtained to assess for clinical toxicity.

The Wilcoxon signed-rank test was used to compare follow-up QOL scores to baseline scores. Decreases in the mean QOL scores at follow-up were considered clinically significant if they were worse by the minimally important difference (MID), which was defined as ½ of the baseline standard deviation (SD) [28]. All tests were two-tailed, and a value of *p* < 0.05 was considered significant. SAS® version 9.2 was used to perform the statistical analyses.

## Results

Overall, we treated 365 patients with SBRT monotherapy and 120 patients with a SBRT boost and IMRT between January 2008 and May 2011. Fifty-one of these patients fit the inclusion criteria for this study, and their characteristics are described in Table 1. The median interval from completion of SBRT to endoscopy was 13 months. The median clinical follow-up was 23 months. Two out of the 51 patients had 2 colonoscopies after SBRT.

**Table 1:** Patient characteristics (n = 51)

**Characteristic**	**Median (range)**
Age	67 (range 52–83)
Length of clinical follow-up (months)	23 (range 8–47)
Time after radiotherapy of the last post-treatment endoscopy (months)	13 (range 1–35)
**Characteristic**	**Number (%)**
Ethnicity	
Caucasian	30 (59%)
African-America	20 (39%)
Other	1 (2%)
Pre-SBRT use of anticoagulation therapy	11 (22%)
Pre-SBRT hemorrhoids	4 (8%)
Radiotherapy	
SBRT monotherapy	35 (69%)
SBRT boost	16 (31%)
Androgen deprivation therapy	
No	35 (69%)
Yes	16 (31%)
NCCN risk category	
Low	14 (27%)
Intermediate	28 (55%)
High	9 (18%)
Gleason score	
6	18 (35%)
7	24 (47%)
8	4 (8%)
9	5 (10%)
T stage	
1c	28 (55%)
2a	10 (20%)
2b	8 (16%)
2c	5 (10%)
PSA	
≤ 10 ng/mL	41 (80%)
> 10 ng/mL	10 (20%)

A total of 16 (31%) patients reported rectal bleeding after SBRT, with 12 (24%) patients reporting acute bleeding and 10 patients (20%) reporting late bleeding. The highest rate of rectal bleeding occurred within 1 month post radiation treatment with 10 patients admitting to either Grade 1 or Grade 2 rectal bleeding. Six of these 10 patients experienced complete symptomatic resolution of these acute bleeds by the subsequent follow-up visit at 3 months. Grade 2 rectal bleeding was observed by only one patient who required minor cauterization secondary to a focal area of bleeding telangiectasias. Four of the 16 patients presenting with rectal bleeding (acute or late) had evidence of hemorrhoids without telangiectasias on endoscopy (see below). Overall, Grade 2 acute and late clinical rectal toxicities were observed in 10 (20%) and 3(6%) of patients, respectively. The majority of the toxicities were observed at one specific follow-up appointment and did not persist on subsequent follow-ups. There were no Grade 3 or higher acute or late clinical rectal toxicities.

**Figure 2:**
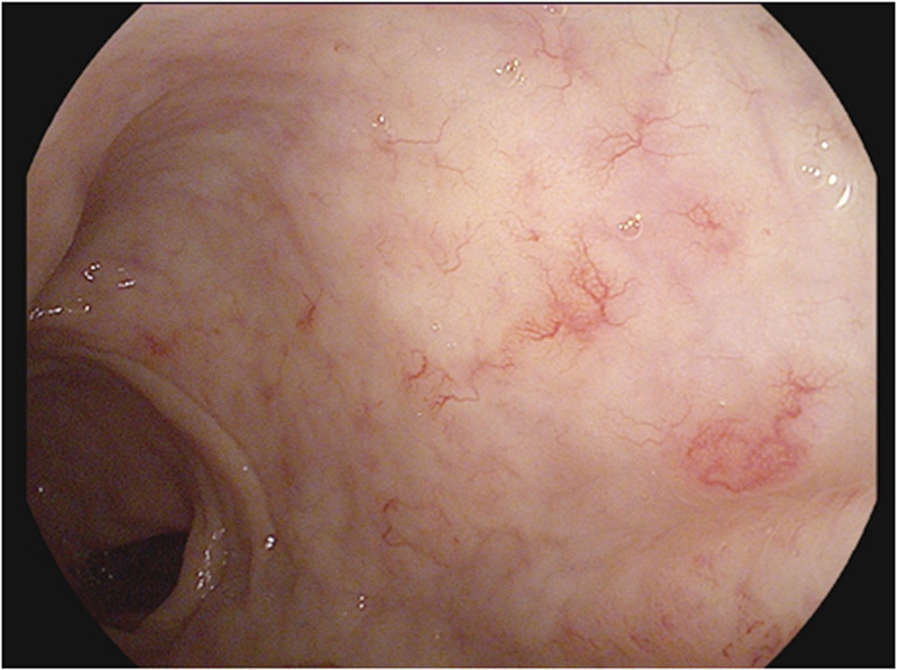
Example of multiple non-confluent telangiectasia (VRS Grade 2) on endoscopy.

On endoscopy, telangiectasias were found in 10 (20%) patients: 6 were treated with SBRT monotherapy and 4 where treated with SBRT as a boost. Nine of the 10 patients were observed to have non-confluent telangiectasias (VRS Grade 2) (Figure 2), and one was observed to have a single telangiectasia (VRS Grade 1). No patient had circumferential telangiectasia. Twelve patients (24%) had evidence of rectal mucositis on endoscopy, 11 patients (22%) with VRS Grade 1 and 1 patient (2%) with VRS Grade 2. No patients had a VRS grade for mucositis or telangiectasia of 3 or higher. No rectal ulcerations, strictures, or fistulas were observed. Of the 10 patients with late rectal bleeding, 3 had evidence of both telangiectasias and mucositis on endoscopy, 1 had telangiectasias without mucositis, and 1 had mucositis without telangiectasias.

Twenty patients (39%) were noted to have hemorrhoids on endoscopy, although only 1 of these patients had complained of clinical symptoms of hemorrhoids on follow-up. Three additional patients had clinical symptoms of hemorrhoids but had no hemorrhoids seen during the time of endoscopy.

**Figure 3:**
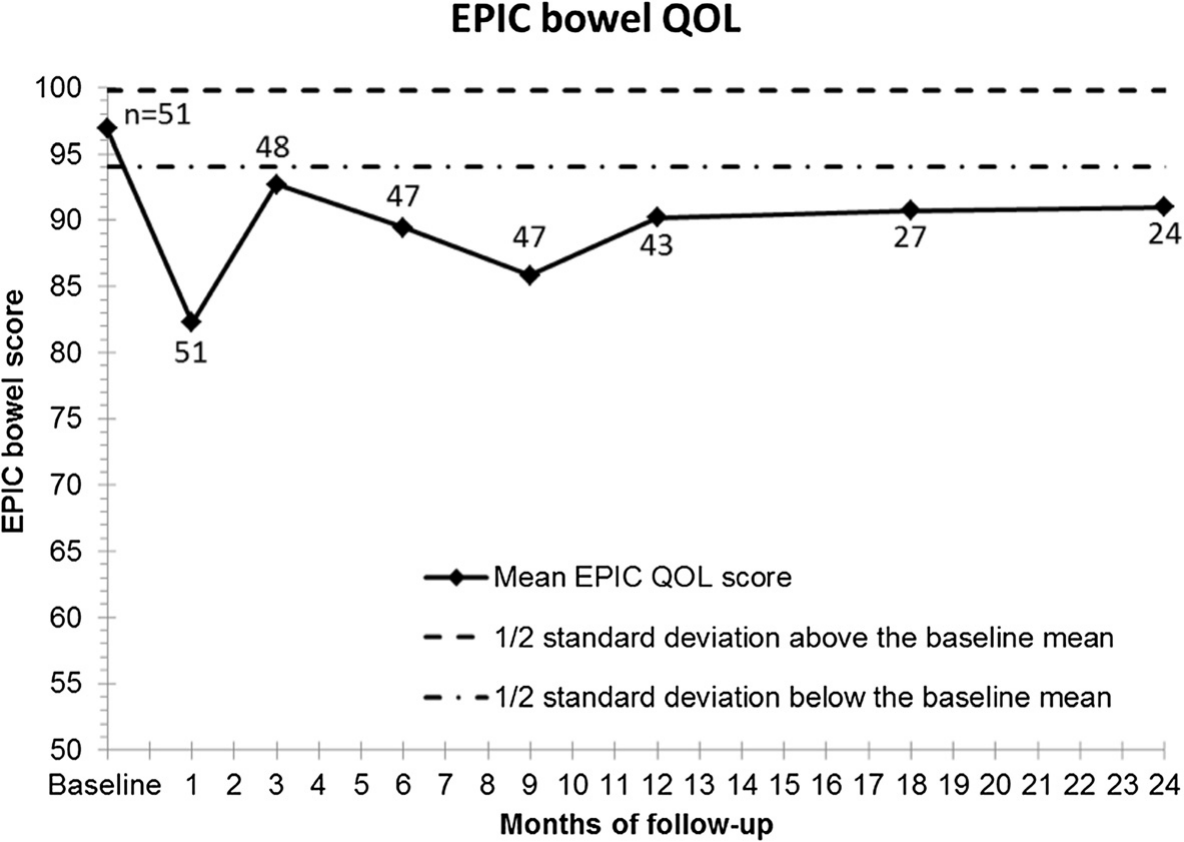
**Changes in EPIC bowel QOL post-SBRT as a function of time.** Mean EPIC bowel score for all patients reporting at the specified time points at baseline and in follow-up. The numbers represent the number of patients answering the questionnaire at that time point. The dashed ½ SD lines represent the clinical minimally important difference.

Mean EPIC bowel scores decreased from a baseline value of 96.9 to 82.3 at 1-month (*p* < 0.001). These values subsequently improved, but were still lower compared to baseline values with 90.2 at 12-months (*p* < 0.001) and 91.0 at 24-months (*p =* 0.01) post-SBRT. Figure 3 illustrates the change of EPIC bowel QOL over subsequent follow-up.

## Discussion

Stereotactic body radiation therapy (SBRT) offers to minimize radiation-associated rectal toxicity by reducing the volume of rectum receiving high radiation doses. The low rate of late Grade ≥ 2 rectal toxicity seen in this study is consistent with the results from a prior review by our institution [19] and results from other institutions, [20-22] which all report a rate of late Grade ≥ 2 rectal toxicity of < 10%. The lack of acute or late Grade 3 toxicity in these cohorts is particularly encouraging. Endoscopy is the gold standard for assessing rectal mucosal injury following radiation therapy. Our study is the first to examine endoscopic outcomes after prostate SBRT.

Since many of the patients in our study had an endoscopy to evaluate rectal symptoms, the study population is skewed towards patients who have more rectal complaints. However, even in this select group of patients, the rate of telangiectasias of 20% is lower than the rate of 32%-88% reported in prospective studies that have looked at endoscopic outcomes after 3D-CRT or IMRT [6,26,29-32]. In addition, no diffuse/confluent (VRS Grade 3) telangiectasias were seen in our patients, while a rate of 5%-25% is reported in the other studies. Based on our results and the results of others, we do not believe that intrarectal amifostine is necessary during prostate SBRT as others have reported [20].

In this select patient population, the overall incidence of post-treatment bleeds is 31%. However, only half of the patients who had telangiectasia on our study had symptomatic rectal bleeding. Additionally, 25% of our patients who reported rectal bleeding had hemorrhoids without evidence of telangiectasias on endoscopy. Together, this suggests that there could be alternative causes for the post-treatment bleeding seen in SBRT-treated patients aside from late post-radiotherapy telangiectasia. These causes could potentially include acute anal irritation or exacerbation of hemorrhoids.

Despite studying a patient population weighted towards those with rectal complaints, the pattern seen in the mean rectal QOL after SBRT in our study is similar to the pattern seen after conventionally fractionated radiotherapy or brachytherapy. The mean QOL score is at its lowest 1 or 2 months after treatment, but improves slowly thereafter to near baseline by 1–2 years after treatment.

Our study is limited by the retrospective nature of the analysis. Another limitation of this study is that the number of patients is relatively small, hampering our ability to perform an analysis of patient-specific and treatment-related factors that can affect rectal toxicity. In addition, the median follow up in this group of patients is relatively short, and additional clinical or endoscopic toxicities could potentially be seen with longer follow up.

## Conclusions

The treatment of prostate cancer with SBRT delivered using the CyberKnife system to doses of 35–36.25 Gy in 5 fractions or 19.5 Gy in 3 fractions with 45–50.4 Gy IMRT has a low rate of Grade > 2 or higher toxicity. No rectal strictures, fistulas, ulcers, or perforations were observed. Prospective trials examining the endoscopic outcomes following SBRT for prostate cancer are needed for confirmation of the findings of this study.

## Abbreviations

SBRT: Stereotactic body radiation therapy; SV: Seminal vesicles; EBRT: External-beam radiotherapy; HDR: High dose-rate; CK: CyberKnife; NCCN: National Comprehensive Cancer Network; GTV: Gross tumor volume; CTV: Clinical target volume; PTV: Planning target volume; QOL: Quality of life; EPIC: Expanded Prostate Cancer Index Composite; CTCAE: Common Terminology Criteria for Adverse Events; VRS: Vienna Rectoscopy Score; 3D-CRT: Three-dimensional conformal radiotherapy; RTOG/EORTC: Radiation Therapy Oncology Group/European Organization for Research and Treatment of Cancer; PSA: Prostate Specific Antigen; RT: Radiotherapy; SD: Standard deviation; MID: Minimal important difference; IMRT: Intensity modulated radiotherapy.

## Competing interests

The Department of Radiation Medicine at Georgetown University Hospital receives an educational grant from Accuray to support a research coordinator. Dr. Sean Collins is a clinical consultant for Accuray.

## Authors’ contributions

SS is the lead author, who participated in the data collection and data analysis, and who wrote the primary drafts. AJ participated in the design of the project, oversaw the data collection and data analysis, and aided in drafting and revising the manuscript. HW is a biostatistician who participated in the statistical analysis and revised the statistical sections of the paper. SL is the dosimetrist who developed the majority of the patients’ treatment plans, and contributed to the dosimetric data analysis and interpretation. SU aided in the quality of life data collection. GZ is a senior author who collected the dosimetric data and participated in its analysis. SS is a senior author who collected the dosimetric data and participated in its analysis. JC and JL are senior authors who aided in drafting the manuscript. AD is a senior author who aided in drafting the manuscript and revising its content. SC was the principle investigator who initially developed the concept of the study and the design, aided in data collection, and helped in revising the manuscript. All authors read and approved the final manuscript.
